# Suspicious CT Findings Suggesting Mediastinitis or Sternal Osteomyelitis in Clinically Uninfected Patients After Cardiac Surgery: A 10-Year Single-Center Retrospective Study

**DOI:** 10.3390/diagnostics16101494

**Published:** 2026-05-14

**Authors:** Maged Makhoul, Lilian Khoury, Noa Leizarowitz, Roi Glam, Tom Friedman, Farouk Khury, Shafra Mubarak, M. Yousuf Salmasi, Gil Bolotin

**Affiliations:** 1 Department of Cardiac Surgery, Rambam Health Care Campus, Haifa 3109601, Israel; noa21688@gmail.com (N.L.); glamroi@gmail.com (R.G.); t_friedman@rambam.health.gov.il (T.F.); g_bolotin@rambam.health.gov.il (G.B.); 2 Clinical Biochemistry Laboratory, Rambam Health Care Campus, Haifa 3109601, Israel; 3 Division of Orthopedic Surgery, Rambam Medical Center, The Ruth and Bruce Rappaport Faculty of Medicine, Haifa 3109601, Israel; 4 Department of Thoracic Surgery, Harefield Hospital, Royal Brompton & Harefield NHS Foundation Trust, Harefield, Middlesex UB9 6JH, UK; shaframubarak@gmail.com (S.M.); y.salmasi@imperial.ac.uk (M.Y.S.)

**Keywords:** CT scan, cardiac surgery, sternal osteomyelitis, mediastinitis

## Abstract

**Background/Objectives**: Post-sternotomy mediastinitis and sternal osteomyelitis are serious complications of cardiac surgery associated with substantial morbidity and mortality. Computed tomography (CT) is widely used to evaluate suspected infection, but the frequency with which CT reports suggest infection in clinically uninfected post-sternotomy patients is poorly characterized. **Methods**: A retrospective observational study was conducted at a tertiary cardiac surgery center. Using an institutional data warehouse, all adult patients undergoing cardiac surgery via median sternotomy between 2010 and 2020 were identified. Patients with documented mediastinitis, sternal osteomyelitis, other postoperative infections, antibiotic treatment, or infectious disease consultation were excluded, as were patients without postoperative CT, those with coronary CT angiography only, and those whose CT scans were performed within 14 days or more than 1 year after surgery. CT reports of the remaining clinically uninfected patients were reviewed and categorized as either showing no evidence of mediastinitis/sternal osteomyelitis or containing findings interpreted as suspicious for these complications. **Results**: Among 4019 patients who underwent cardiac surgery during the study period, 92 highly selected clinically uninfected adults met the inclusion criteria and had eligible postoperative CT scans. Of these, 60 had coronary artery bypass grafting, 6 had mitral valve replacement, 17 had aortic valve replacement, and 9 had ascending aortic replacement. Four patients (4.4%; 95% CI, 1.2–10.9%) had CT reports describing findings suggestive of mediastinitis and/or sternal osteomyelitis despite the absence of concomitant clinical or laboratory evidence of infection. All four were post-coronary artery bypass grafting patients and had common radiologic features reported in postoperative infection, including sternal edge irregularity/erosion, sclerosis, retrosternal fluid collections, and mediastinal or presternal fat stranding. **Conclusions**: In this single-center retrospective series, CT reports suggesting mediastinitis or sternal osteomyelitis were observed in a small proportion of carefully selected, clinically uninfected post-sternotomy patients. These findings support the need to interpret CT abnormalities after cardiac surgery in close conjunction with clinical and laboratory data to avoid unnecessary invasive interventions in patients without true infection.

## 1. Introduction

Median sternotomy has remained the standard surgical approach for most cardiac procedures since the 1950s. Post-sternotomy mediastinitis and sternal osteomyelitis are feared complications associated with prolonged hospitalization, re-interventions, and high mortality, despite advances in perioperative care and infection control. Reported incidence rates of mediastinitis after cardiac surgery range from approximately 0.3% to 3.4%, with in-hospital mortality in affected patients reaching 20% in some series [1,2].

Clinical diagnosis of post-sternotomy mediastinitis or sternal osteomyelitis can be challenging. Early postoperative findings such as pain, low-grade fever, and superficial wound changes are common and nonspecific. Reliance on clinical signs alone may therefore be insufficient to differentiate between normal postoperative recovery, superficial wound problems, and deep sternal infection. In this setting, imaging has become an essential adjunct to clinical assessment and frequently informs decisions regarding antibiotic initiation, surgical revision, and sternal reconstruction [1,3].

**Figure 1:**
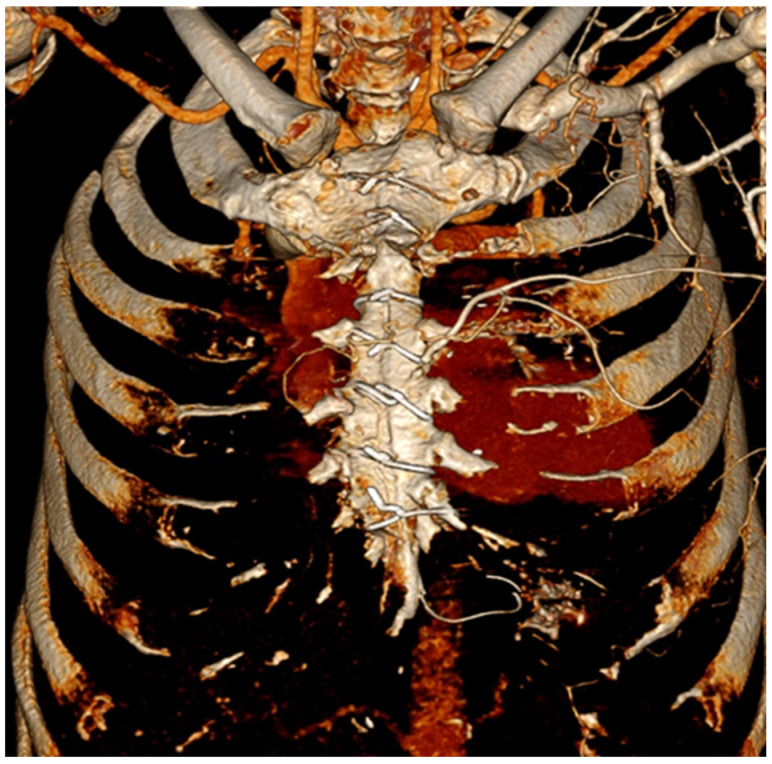
Three-dimensional reconstruction of a CT scan of the sternum after sternotomy. We ask: is it infected?

CT is currently the most widely used imaging modality for evaluating suspected postoperative mediastinitis and sternal osteomyelitis after cardiac surgery [4,5]. Reported sensitivity and specificity vary widely, from 25% to 100% and 33% to 100%, respectively, and are strongly influenced by timing after surgery and by the radiologic criteria used. Before postoperative day 14, CT shows high sensitivity but limited specificity because postoperative collections, edema, hematomas, and gas can mimic infection. Beyond 2–3 weeks, the specificity of CT has been reported to improve substantially as early postoperative changes resolve and sternal healing progresses [3,4,6] (Figure 1).

However, the sternum after median sternotomy undergoes a complex healing process that may itself resemble pathologic changes on CT [5,7]. The osteotomy is created with a powered saw, leaving sharp, sclerotic cortical margins. Bone healing proceeds through periosteal and endosteal callus formation, with gradual bridging and eventual fusion of the sternal halves. Importantly, radiologic union often lags behind clinical union, and CT may underestimate the degree of sternal stability and healing. Radiologic features of osteomyelitis typically include radiolucent cortical or medullary lesions with surrounding sclerosis, sometimes described as localized abscesses. These features can overlap with expected postoperative osteotomy remodeling, particularly along sclerotic osteotomy margins [5,7,8].

Given this overlap, there is a theoretical risk that normal or slowly healing sternotomy changes may be misinterpreted as osteomyelitis or mediastinitis on CT, especially when interpreted without a full clinical context. Despite this concern, the literature contains limited data on the frequency of CT reports suggesting mediastinitis or sternal osteomyelitis in patients who are clinically uninfected after cardiac surgery.

This study aimed to quantify the proportion of postoperative CT reports with findings suggestive of mediastinitis or sternal osteomyelitis in a cohort of adult post-sternotomy patients without clinical, laboratory, or microbiological evidence of infection, thereby addressing a critical and underexplored source of diagnostic uncertainty. In addition, we sought to characterize the radiologic features and clinical profiles of these patients.

## 2. Materials and Methods

### 2.1. Study Design and Setting

This was a retrospective observational study conducted at a tertiary medical center with a medium-volume cardiac surgery program and on-site CT imaging facilities. The study period extended from January 2010 to December 2020, and the patients were enrolled consecutively. The institutional review board approved the study protocol, including a waiver of informed consent (approval number 0326-20-RMB, approval date 13 August 2020), given its retrospective design and use of anonymized data.

### 2.2. Patient Identification and Data Sources

All adult patients (≥18 years) who underwent cardiac surgery via median sternotomy during the study period were identified using an institutional big-data platform (MDClone^®^, Beer-Sheva, Israel) and the hospital’s electronic medical records. Demographic, clinical, operative, microbiological, and imaging data were extracted from the electronic health record.

### 2.3. Inclusion Criteria

**Figure 2:**
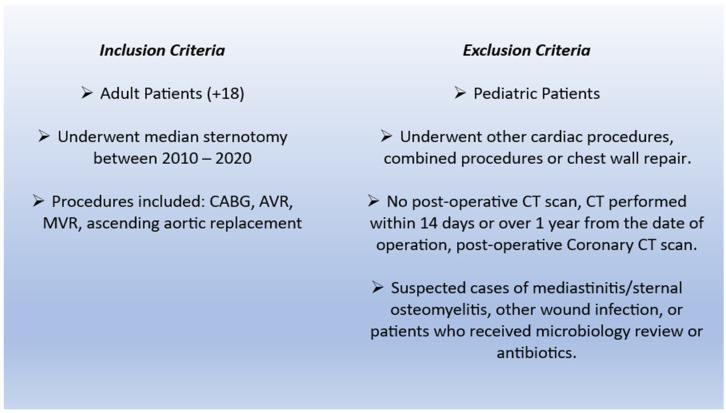
Inclusion and Exclusion Criteria. CT-computed tomography, CABG-coronary artery bypass grafting, AVR-aortic valve replacement, MVR-mitral valve replacement.

Patients were eligible for analysis if they met all of the following criteria (Figure 2):

- Age ≥ 18 years at the time of surgery.
- Underwent one of the following index procedures via median sternotomy: coronary artery bypass grafting (CABG), aortic valve replacement (AVR), mitral valve replacement (MVR), or ascending aortic replacement.
- Had at least one postoperative thoracic CT scan (standard chest CT with or without contrast or CT performed for pulmonary embolism protocol) performed between 14 days and 1 year after the index operation.

### 2.4. Exclusion Criteria

The following exclusion criteria were applied sequentially (Figure 2):

- Pediatric patients (age < 18 years).
- Patients who underwent other cardiac procedures, combined procedures, or chest wall repair.
- Documented mediastinitis or sternal osteomyelitis during the index hospitalization or follow-up period, based on clinical, microbiological, or surgical data.
- Any postoperative infection, including those evaluated by infectious disease or microbiology consultation or treated with systemic antibiotics, irrespective of site.
- Absence of a postoperative thoracic CT scan.
- CT scans performed within 14 days of surgery or more than 1 year after surgery.
- Postoperative coronary CT angiography as the only CT examination, because coronary protocols are not optimized for detailed assessment of the sternum and mediastinum, and because the interpretation did not include assessment of the sternum and the mediastinum.
- CT scans with documented suspicion of mediastinitis or sternal osteomyelitis in patients who ultimately received antibiotic therapy or underwent surgical re-exploration for infection.

To classify patients as “clinically uninfected”, the following were reviewed: clinical notes (including cardiac surgery and infectious disease consultations), vital signs, laboratory tests (including white blood cell count and C-reactive protein), microbiologic cultures, antibiotic prescriptions, and operative or procedural reports.

Perioperative prophylactic antibiotic administration was not considered an exclusion criterion. Patients who received systemic antibiotic therapy beyond standard prophylaxis for suspected or confirmed infection were excluded. No predefined cut-off values for inflammatory markers (e.g., white blood cell count or C-reactive protein) were used; instead, classification was based on comprehensive clinical assessment, including trends in laboratory parameters in conjunction with clinical and microbiological findings.

### 2.5. Definitions

Mediastinitis and sternal osteomyelitis were defined according to the Centers for Disease Control and Prevention (CDC) criteria for healthcare-associated infections and contemporary literature [9]. Mediastinitis required at least one of the following: (1) organisms identified from mediastinal tissue or fluid by culture or appropriate non-culture-based methods; (2) evidence of mediastinitis on gross anatomic or histopathologic examination; or (3) a compatible clinical syndrome (chest pain, sternal instability, or fever > 38 °C) in combination with purulent mediastinal drainage or isolation of an organism from blood culture or mediastinal specimens. Post-sternotomy mediastinitis was additionally considered a deep sternal wound infection involving the sternum (sternal osteomyelitis) with or without retrosternal space involvement, occurring within 1 year of the index operation.

For the purposes of this study, patients who did not meet CDC criteria, did not develop any clinical, laboratory, or microbiological evidence of infection during the period up to at least 1 year after surgery (or until last available follow-up within that year), and did not receive targeted antibiotic therapy for suspected mediastinitis/sternal osteomyelitis were classified as uninfected.

### 2.6. CT Acquisition and Reporting

Eligible CT scans included standard chest CT examinations with or without intravenous contrast and CT studies performed with pulmonary embolism protocols. Coronary CT angiography studies were excluded. CT examinations were performed according to institutional protocols using multidetector scanners. All CT reports were generated in routine clinical practice by radiologists and signed by at least a senior/consultant radiologist. For this study, the original CT reports were retrieved and interpreted as they were at the time of clinical care; no additional blinded rereading was performed.

CT reports were reviewed and categorized into two groups:

Group A: no reported findings suggestive of mediastinitis or sternal osteomyelitis.

Group B: reports describing findings interpreted in the text as suspicious for mediastinitis and/or sternal osteomyelitis (e.g., sternal edge erosion or destruction, cortical irregularity, sclerosis with lucencies, retrosternal or presternal fluid collections, mediastinal fat stranding, or explicit mention of possible osteomyelitis or mediastinitis).

### 2.7. Data Collection and Validation

For all included patients, demographic variables (age, sex), index procedure type, major comorbidities (including peripheral vascular disease, chronic obstructive pulmonary disease, diabetes mellitus, hyperlipidemia, and hypertension), and use of internal mammary artery (IMA) grafts were recorded. For CT examinations, the type of CT (standard chest CT versus pulmonary embolism protocol), indication for the scan, timing in days from surgery, and the main reported sternal and mediastinal findings were extracted.

To minimize classification errors, medical and radiologic records of all included patients were independently reviewed by two physicians with experience in cardiac surgery and intensive care. Discrepancies were resolved by consensus.

### 2.8. Flow-Up Data

Follow-up data were obtained by reviewing the medical records of the six patients in Group B, including clinical reports, laboratory results, and imaging studies, during manuscript preparation (August 2025). Follow-up time was defined as the interval from the date of surgery to the end of the study period (1 August 2025). Follow-up duration was calculated in months and summarized using the median and interquartile range (IQR).

The one-year timeframe was used as an inclusion criterion for CT acquisition, not as a strict limit for outcome assessment. Patients in Group A, who had no clinical or radiologic suspicion of infection, were not subject to extended follow-up beyond routine clinical documentation. In contrast, patients in Group B underwent extended follow-up through detailed review of medical records up to August 2025 to exclude delayed or indolent infection.

### 2.9. Statistical Analysis

Given the exploratory nature and limited sample size, the analysis was descriptive. Categorical variables are presented as counts and percentages, and continuous variables as means with standard deviations or medians with interquartile ranges, as appropriate. The primary outcome was the proportion of clinically uninfected patients with postoperative CT scans whose reports contained findings suggestive of mediastinitis or sternal osteomyelitis (Group B). No formal hypothesis testing was performed.

## 3. Results

### 3.1. Study Population and CT Eligibility

**Figure 3:**
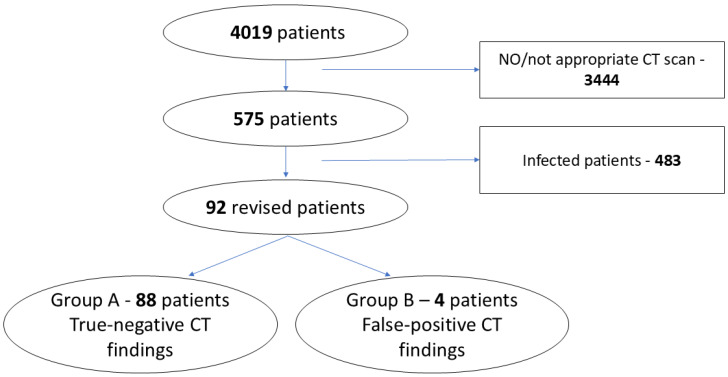
Flowchart describing the patient selection. CT-computed tomography.

During the 10-year study period, 4019 adult patients underwent cardiac surgery via median sternotomy at the study center. Of these, 142 patients (3.5%) had documented mediastinitis or sternal osteomyelitis and were excluded. An additional 341 patients had other postoperative infections or received systemic antibiotic treatment and were also excluded. Patients without any postoperative thoracic CT scan (n = 3197) or with coronary CT angiography only (n = 166) were not eligible for analysis. Eighty-one patients were excluded because their CT scan was performed either within 14 days or more than 1 year after surgery. After application of all inclusion and exclusion criteria, 92 clinically uninfected adult patients with eligible postoperative CT scans remained for analysis. (Figure 3).

### 3.2. Baseline Characteristics

Among the 92 included patients, 61 (66%) were male and 31 (34%) were female. The index procedures were CABG in 60 patients, MVR in 6 patients, AVR in 17 patients, and ascending aortic replacement in 9 patients. Common comorbidities included hypertension, hyperlipidemia, and diabetes mellitus, consistent with a typical contemporary cardiac surgery population (Table 1).

### 3.3. CT Findings Suggestive of Infection in Uninfected Patients

Of the 92 clinically uninfected patients, 88 (95.6%) had CT reports without findings suggestive of mediastinitis or sternal osteomyelitis (Group A), whereas 4 patients (4.4%; 95% CI, 1.2–10.9%) had CT reports describing features interpreted as suspicious for these complications (Group B). All four patients in Group B had undergone CABG and received a single internal mammary artery graft; none had bilateral IMA harvesting.

The prevalence of common comorbidities in Group B, including diabetes mellitus and peripheral vascular disease, was broadly comparable to that observed in the overall cohort, and no distinct clinical pattern was identified. Given the small sample size, no formal statistical comparisons were performed.

In Group B, 1 patient (25%) had peripheral vascular disease and 3 (75%) had diabetes mellitus, while all four had hypertension and hyperlipidemia; none had chronic obstructive pulmonary disease. These comorbidity profiles placed them at low to moderate risk for deep sternal wound infection but did not represent an extremely high-risk subgroup.

The indications for CT in these four patients included chest pain, evaluation for pulmonary embolism, and atypical chest symptoms. Reported CT features encompassed sternal non-union or gapping with sclerotic margins, irregular or eroded sternal borders, retrosternal fluid collections, and mediastinal or presternal fat stranding, sometimes described as potentially consistent with osteomyelitis or chronic infection (Table 2). Despite these radiologic descriptions, none of the patients had concurrent clinical or laboratory signs of infection, none received targeted antibiotic therapy for mediastinitis or sternal osteomyelitis, and none underwent surgical re-exploration for sternal infection during the defined postoperative period. During the follow-up period, no cases of mediastinitis or sternal osteomyelitis were observed among patients in Group B. The median follow-up time was 133.5 months (IQR: 99–152 months).

Patients in Group B were followed through detailed chart review until August 2025 to exclude delayed or indolent infection.

## 4. Discussion

In this single-center retrospective study of adult post-sternotomy patients without clinical, laboratory, or microbiological evidence of infection, CT reports describing findings suggestive of mediastinitis or sternal osteomyelitis were observed in 4 of 92 patients (4.4%; 95% CI, 1.2–10.9%) who underwent postoperative thoracic CT between 14 days and 1 year after cardiac surgery. These patients were clinically uninfected, did not receive targeted antibiotic treatment, and did not undergo surgical interventions for deep sternal wound infection, indicating that the suspicious CT features did not correspond to clinically manifest mediastinitis or sternal osteomyelitis during the follow-up period.

The most commonly reported CT features in these cases—sternal edge irregularity or erosion, sclerosis with apparent gaps, retrosternal fluid collections, and mediastinal or presternal fat stranding—are the same elements frequently cited as radiologic signs of postoperative mediastinitis and osteomyelitis in the literature. However, similar changes may also arise from expected postoperative healing, osteotomy remodeling, and localized reactions around sternal wires [10,11,12,13]. The overlap between normal postoperative appearances and infection-related changes creates a gray zone in CT interpretation, particularly when scans are performed in the subacute postoperative window.

Although computed tomography represents the cornerstone imaging modality for the evaluation of suspected post-sternotomy mediastinitis, a multimodality imaging approach could be considered in selected equivocal cases. In particular, positron emission tomography may provide additional functional information that could help differentiate active infection from postoperative inflammatory changes; however, the available evidence in this specific setting remains limited and heterogeneous. In rare situations where there is concern for cardiac involvement, echocardiography may be useful to exclude associated intracardiac pathology. Overall, integration of imaging findings with clinical and laboratory data remains essential to improve diagnostic accuracy and avoid misinterpretation, particularly in light of the false-positive CT findings observed in our cohort [14].

Existing studies evaluating CT in post-sternotomy patients have largely focused on patients with suspected infection and have reported relatively high sensitivity and variable specificity, especially when CT is performed more than 2–3 weeks after surgery [8,15]. In contrast, few data describe how often CT reports raise suspicion of deep infection in patients who are otherwise clinically uninfected [4]. The current study offers an initial estimate in a highly selected cohort. It highlights that such radiologic concerns, although infrequent, do occur and can potentially prompt unnecessary anxiety, investigations, or interventions if not weighed carefully against the overall clinical picture.

The risk profile of the four patients with suspicious CT findings in this series was not markedly different from the broader cardiac surgery population. All had typical cardiovascular comorbidities; although diabetes and peripheral vascular disease are recognized risk factors for deep sternal wound infection, the absence of other high-risk features such as bilateral IMA harvesting or severe chronic obstructive pulmonary disease suggests that these patients were not at the end of the risk spectrum. This supports the interpretation that the suspicious CT features likely reflected benign or slowly healing postoperative changes rather than unrecognized clinical infection.

From a clinical perspective, these findings highlight that CT abnormalities after sternotomy should not be interpreted in isolation. Although CT is widely regarded as a sensitive modality for detecting mediastinitis, its specificity remains variable, particularly in the subacute postoperative period when normal healing changes may mimic infection. Our results support existing literature by demonstrating that radiologic features commonly associated with infection, such as sternal irregularity, sclerosis, or small retrosternal collections, may also be present in clinically uninfected patients. Therefore, CT should not be considered a standalone diagnostic arbiter of post-sternotomy mediastinitis or sternal osteomyelitis. Instead, imaging findings must be interpreted within the broader clinical context, incorporating clinical assessment, laboratory markers, and microbiologic data. In patients with low clinical suspicion and stable postoperative courses, such findings should be approached with caution, particularly when CT is performed for unrelated indications (e.g., evaluation for pulmonary embolism). These considerations underscore the importance of a structured, multidisciplinary approach to diagnosis, as well as the potential value of standardized reporting frameworks that distinguish expected postoperative changes from findings more suggestive of true infection, thereby improving communication and reducing the risk of unnecessary interventions.

Nevertheless, although certain CT features have been associated with postoperative infection in prior studies, there is currently no validated or widely accepted CT-based scoring system to reliably distinguish normal postoperative changes from true mediastinitis or sternal osteomyelitis. Given the limited number of cases with suspicious findings in our cohort, we were not able to derive or validate diagnostic criteria, and therefore no specific imaging thresholds can be recommended based on the present data.

This study has several important limitations. It was retrospective and single-center, with a relatively small number of included patients and only four cases with suspicious CT reports, which limits the precision and generalizability of the estimated proportion. The denominator consisted of patients who underwent postoperative thoracic CT for various clinical reasons, introducing selection and indication bias; therefore, the results may not reflect the behavior of CT in all post-sternotomy patients. The very small number of patients in Group B limited the ability to explore potential risk factors or perform meaningful statistical comparisons. No centralized blinded rereading of CT scans was performed, and the analysis relied on original clinical reports, which may vary in style and threshold for labeling findings as suspicious. In addition, laboratory markers such as white blood cell count and CRP were recorded quantitatively but interpreted qualitatively based on their temporal trends and overall clinical context rather than predefined numerical thresholds. While this approach reflects real-world clinical practice in postoperative cardiac surgery patients, it may limit reproducibility and standardization across different settings.

Finally, although patients were classified as uninfected based on a comprehensive chart review and follow-up within 1 year, subclinical or very late infections beyond this window cannot be completely excluded. In addition, the definition of ‘clinically uninfected’ was based on comprehensive clinical assessment rather than predefined laboratory thresholds or standardized criteria for inflammatory markers and antibiotic use, which may limit reproducibility across different settings.

The extensive inclusion and exclusion criteria resulted in a highly selected cohort representing a small fraction of the overall post-sternotomy population. While this approach was necessary to isolate clinically uninfected patients and better estimate false-positive CT findings, it may limit the generalizability of the observed 4.4% rate to broader clinical settings, particularly in patients with intermediate pretest probability of infection. Therefore, these findings should be interpreted within the context of this selected population.

Moreover, CT reports were generated in routine clinical practice by radiologists with access to clinical information, which may have influenced interpretation; however, given that the included patients were clinically uninfected and lacked suspicion for deep sternal infection, such bias is unlikely to have systematically affected the results.

Despite these limitations, the study underscores that CT findings interpreted as suggestive of mediastinitis or sternal osteomyelitis can be encountered even in carefully vetted, clinically uninfected post-sternotomy patients. Awareness of this phenomenon may help multidisciplinary teams avoid overdiagnosis and overtreatment while still recognizing genuine infections in patients with compatible clinical presentations.

## 5. Conclusions

In this retrospective single-center study, CT demonstrated a low but notable rate of false-positive findings suggestive of mediastinitis or sternal osteomyelitis in clinically uninfected post-sternotomy patients. These findings should be interpreted with caution, particularly in the absence of supporting clinical or laboratory evidence. Given the limited sample size and study design, further studies are warranted to better define the diagnostic accuracy of CT and to optimize imaging-based decision-making in this setting.

## Figures and Tables

**Table 1:** Clinical characteristics of patients with CT findings suggestive of infection (Group B, n = 4).

Patient Nr	1	2	3	4
Age/Sex	62/F	63/M	58/F	57/M
Cardiac procedure	CABG	CABG	CABG	CABG
Operation date	3 January 2012	21 October 2013	9 February 2015	21 July 2019
CT-timing (Days after surgery)	126	36	30	40
PVD	No	Yes	No	No
COPD	No	No	No	No
Diabetes mellitus	No	Yes	Yes	Yes
hyperlipidemia	Yes	Yes	Yes	Yes
Hypertension	Yes	Yes	Yes	Yes
IMA used	1	1	1	1

CT—computed tomography; PVD—peripheral vascular disease; COPD—chronic obstructive pulmonary disease; CABG—coronary artery bypass grafting; IMA—internal mammary artery.

**Table 2:** CT type, indications, and reported findings in patients with suspicious CT features (Group B, n = 4).

Patient Nr	CT Type	Indication for CT	CT Findings
1	CT chest	Chest pain	Sternal gaping is consistent with nonunion. Additionally, there is sclerosis of both sternal borders. Without significant parasternal fat stranding. Osteomyelitis of the sternum cannot be ruled out though the absence of major inflammatory signs
2	CT chest	Pulmonary embolism	Retrosternal fluid collection of up to 2 cm. Ill-defined, and irregularity of sternal borders—in alliance with surgical margins—might be inflammatory.
3	CT chest	Atypical chest pain.	Erosion and irregularity of sternotomy borders—suspected as osteomyelitis. Focal mild retrosternal fluid collection and fat stranding are consistent with inflammatory/infectious processes surrounding the sternum.
4	CT-PE protocol	Pulmonary embolism	Sternotomy post-CABG. There is sclerosis and erosions of the sternal borders. Pre-sternal fat stranding observed. The overall imaging findings may be consistent with chronic osteomyelitis.

CT—computed tomography; PE—pulmonary embolism; CABG—coronary artery bypass graft.

## Data Availability

The datasets used and analyzed during the current study available from the corresponding author on reasonable request.

## Abbreviations

The following abbreviations are used in this manuscript:

CT	Computed tomography
CABG	Coronary artery bypass graft
MVR	Mitral valve replacement
AVR	Aortic valve replacement
CDC	Centers for disease control and prevention
PVD	Peripheral vascular disease
COPD	Chronic obstructive pulmonary disease
IMA	Internal mammary artery
PE	Pulmonary embolism
